# ITIH2 in colorectal cancer metastasis: Weighted Gene Co-expression Network Analysis-guided functional validation

**DOI:** 10.1371/journal.pone.0329719

**Published:** 2026-02-05

**Authors:** Xin-Feng Zhang, Xiao-Li Zhang, Hai-Wen Xu, Ya-Hui Lin, Yan Tian, Cheng Chen, Huayou Luo

**Affiliations:** 1 Department of Gastrointestinal and Hernia Surgery, The First Affiliated Hospital of Kunming Medical University, China; 2 Trauma Center, The First Affiliated Hospital of Kunming Medical University, China; University of Helsinki: Helsingin Yliopisto, FINLAND

## Abstract

**Objective:**

One of the main causes of death from colorectal cancer (CRC) is liver metastases; yet, little is known about the genetic factors that influence the development of these metastases. Given their proven involvement in cancer spread, we anticipated that major differentially expressed genes (DEGs) between primary and metastatic CRC could identify important molecular players in liver metastasis, with an emphasis on extracellular matrix and proteolytic processes. The purpose of this study was to use integrative bioinformatics and experimental validation to identify and functionally describe hub genes linked to CRC liver metastasis.

**Methods:**

The differentially expressed genes (DEGs) between primary (CRC and CRC liver metastases are investigated using two microarray datasets (GSE14297 and GSE6988). Weighted Gene Co-expression Network Analysis (WGCNA) was used to generate co-expression networks and identify functional modules associated with metastatic progression. Topological research revealed that ITIH2 is an essential hub gene. To investigate its functional role, we employed lentiviral transduction to generate stable CRC cell lines with ITIH2 overexpression and knockdown. For *in vivo* validation, liver metastasis models were created in BALB/c mice using engineered cell lines. To further explore clinical relevance, ITIH2 expression in tissue microarrays from patients with primary and metastatic CRC was examined immunohistochemically.

**Results:**

When compared to primary tumors, ITIH2 expression was noticeably higher in CRC liver metastases. In contrast to ITIH2 knockdown, which inhibited these malignant behaviors, functional studies showed that ITIH2 overexpression increased CRC cell proliferation, motility, and invasion. These results were supported *in vivo*, where ITIH2 expression increased the likelihood of tumor growth and metastasis.

**Conclusions:**

ITIH2 plays a functional role in metastatic behavior and is markedly elevated in liver metastases of CRC. We conclude that ITIH2 may be a novel prognostic biomarker and a potential therapeutic target in patients with CRC who have liver metastases due to its high correlation with poor clinical outcomes and independent prognostic significance for overall survival.

## 1 Introduction

CRC is the second most common kind of cancer and a leading cause of cancer-related death worldwide [1]. Even when the disease is detected at an advanced stage, the prognosis for patients remains poor [2]. The primary cause of cancer-related death in CRC is metastasis to distant organs such as the liver and lungs. Metastatic tumors exhibit cellular heterogeneity similar to that of the original tissues [3]. Patients with localized CRC now have a 5-year overall survival (OS) rate of over 90% thanks to advancements in complete treatment. On the other hand, less than 15% of CRC patients with distant metastases still have a dismal 5-year OS rate. [4]. Despite increasing advancements in treatment, the mortality rate from CRC, particularly metastatic CRC, remains high among cancer-related deaths [5]. The metastatic cascade is a multi-step cellular biological process that includes cell migration, invasion, metastatic seeding, and proliferation [6]. However, current research has not yet revealed specific gene alterations connected to metastatic CRC, and the underlying mechanisms remain poorly understood [7]. Thus, figuring out the hub gene that plays a role in CRC liver metastasis could help us better understand the pathophysiology and course of the disease and make it easier to find possible treatment targets.

The ability to employ bioinformatics tools to examine the genetic variations between primary CRC and liver metastases has been made possible by the continuous improvement of bioinformatics technology in recent years. Transcriptome data from the GSE14297 and GSE6988 datasets, which include samples from people with primary CRC and those with liver metastases from the disease, are available in the GEO database (https://www.ncbi.nlm.nih.gov/geo/). The inclusion of matched liver metastases and paired samples from primary CRC gives them a distinct advantage. By reducing inter-individual variability, this paired-sample strategy makes it possible to precisely identify changes in gene expression that are directly linked to the metastatic process. Furthermore, the genes we found are more resilient thanks to the cross-validation process that uses two separate datasets. WGCNA is a systems biology method that finds hub genes linked to particular phenotypes by grouping genes into co-expression modules. After being discovered by WGCNA, the hub gene’s biological function was examined by experimental testing. We also acquired transcriptome data for colon cancer from the TCGA database(https://gdc.cancer.gov/about-gdc) to evaluate the predictive importance of the hub gene in the disease. Furthermore, we established stable cell lines with ITIH2 overexpression and knockdown using CCK-8 cell viability assays, Transwell migration assays, and invasion assays. We next assessed the impact of ITIH2 expression on CRC cell migration, invasion, and proliferation *in vitro* and *in vivo*. Our findings demonstrated that whereas downregulating ITIH2 had the opposite impact, upregulating ITIH2 expression enhanced CRC cell invasion, migration, and proliferation *in vitro*. Immunohistochemical testing revealed that ITIH2 expression was significantly higher in patients with CRC liver metastases than in patients with CRC. Our findings suggest that ITIH2 may have a role in the formation of liver metastases from CRC and may potentially promote the disease’s growth and dissemination.

## 2 Materials and methods

### 2.1 Download of materials

The GEO database was used to obtain the gene expression datasets GSE6988 (platform GPL6370) and GSE14297 (platform GPL4811) for this investigation. Because of their distinctive inclusion of paired tissue samples from the same patient (primary CRC and matched liver metastases), which reduces inter-patient variability and enables direct identification of metastasis-associated genes, these two datasets were especially chosen for our research. The data from the GSE6988 and GSE14297 datasets are shown in Table 1. The transcriptome data for colon cancer was then retrieved from the TCGA database in order to evaluate and validate the predictive significance of ITIH2 in colon cancer. In order to confirm the expression of ITIH2 in individuals with CRC and liver metastases, GSE92914 was utilized as an external dataset.

**Table 1:** Details of the two dataset clusters.

Serial number	Platform	Tissue Source	Patient group	Control group	Total number
GSE14297	GPL6370	Primary CRC Liver metastasis	18	18	36
GSE6988	GPL4811	Primary CRC Liver metastasis	29	53	82

### 2.2 Screening for differential genes

Utilizing a modified threshold screening for |log2 FC | > 1.0 and *P* < 0.05. Using the GSE14297 and GSE6988 datasets, which were produced by taking the intersection of the two sets of differential genes (DEGs), we looked for DEGs between patients with primary CRC and patients with liver metastases. Next, we used the WGCNA algorithm to build co-expression networks and DEGs modules between primary CRC and CRC liver metastases between the GSE14297 and GSE6988 datasets.

### 2.3 Protein interaction network analysis of DEGs

To conduct additional analysis, the 89 DEGs that overlapped in the aforementioned list were imported into the STRING database (http://string-db.org/). in order to obtain the list of protein mediators. Cytoscape software is used to view and analyze the PPI network; an interaction value of 0.4 or above was deemed significant. PPI sub-networks were constructed by utilizing the Cytoscape plugin Minimal Common Oncology Data Elements (MCODE) to look for important protein expression molecules. The hub genes in the highly linked PPI network were screened using the LAC algorithm in the CytoNCA plugin. Examining the 20 genes where both plugins were discovered to overlap was the final stage.

### 2.4 Prognostic and diagnostic potential of hub genes

We examined the 20 hub genes’ prognosis. It was found that the overall survival (OS) of patients with colon cancer was predicted by both high and low expression levels of ITIH2. The OS of patients with colon cancer was not predicted by the remaining 19 genes. ITIH2 expression was useful in the diagnosis of patients with liver metastases from CRC. The optional ITIH2 threshold value was used to classify the samples from the TCGA database as either high or low expression. Each patient underwent a Kaplan-Meier (KM) survival analysis to evaluate the OS difference between the two groups. Every colon cancer patient in the database assessment had their Recurrence Free Survival Time (RFS) examined using the internet database GEPIA2 (http://gepia2.cancer-pku.cn/).

### 2.5 Cell lines and culture

Human CRC cell lines SW480, SW620, Caco2, and NCI-H508 were supplied by Prosperity Life Sciences Co. Ltd. (Wuhan, China); the NCI-H508 cells were cultured in RPMI-1640 (Gibco, Carlsbad, CA, USA), while the SW480, SW620, and Caco2 cells were cultured in DMEM (Pricella, Wuhan, China) as their basal medium. 10% FBS and 1% P/S were added to the basal medium, and the cells were then grown at 37°C in an incubator with 5% CO_2_.

### 2.6 Establishing transfection stable strains

HanBio (Shanghai, China) provided our lentiviruses, which overexpress and knock down the ITIH2 gene. Details about the gene sequences and plasmid vectors are provided in Supplementary Table 1 in S1 File. Caco2 and NCI-H508, which had the highest expression levels, were used to establish stable cell lines with ITIH2 knockdown, while SW480 and SW620 were used to create stable cell lines with ITIH2 overexpression. The cell line screening studies are shown in Supplementary Figure1. Once the cell density surpassed 90%, the empty vector virus and the experimental virus strains for overexpression and knockdown were transfected into the corresponding cells in accordance with the manufacturer’s instructions and the cells’ MOI value (MOI × (cell count/viral titer) to calculate the viral volume). For ensuing functional tests, tumor cells were isolated 72 hours following transfection. Three sequences were present in the lentiviral particles containing GFP-PuroITIH2 short hairpin RNA (shRNA) (HanBio, Shanghai, China). We chose one for further experimental research after a successful validation.

### 2.7 Quantitative reverse transcriptase‑polymerase chain reaction (qRT‑PCR)

Total cellular RNA was extracted from cells using the TRIzol reagent (Invitrogen) as directed by the manufacturer. After that, cDNA was reverse-transcribed into cDNA using the All-In-One 5X RT Master Mix (abm, Vancouver, Canada) as directed by the manufacturer. Following that, cDNA was transformed into cDNA for qPCR using a Roche LightCycler® 480 System (Roche, Indianapolis, USA) and the PrimeScriptTM RT reagent Kit (Chengdong District, Osaka City, Japan). The conditions for amplification were as follows: Once, at 95°C for 30 seconds. 40 cycles of 5 s at 95°C and 30 s at 60°C. The relative expression of each mRNA was ascertained using the 2^-ΔΔCt^ approach after the mRNA levels were normalized to those of GAPDH. The primer sequences are listed in Supplemental Table 2 in S1 File.

### 2.8 Protein extraction and western blot

CRC cells were used to isolate total cellular proteins using a radioimmunoprecipitation assay solution (RIPA buffer; Servicebio, Wuhan, China) that contained protease and phosphatase inhibitors. Following that, the cells underwent two 15-minute washes with frozen PBS. The samples were centrifuged at 12,000 rpm for 15 minutes at 4°C, and the protein content was then measured using the bicinchoninic acid (BCA) protein assay kit (Yamay Biomedical Technology Co., Ltd., Shanghai, China). After then, the supernatant was gathered. Protein samples (20 µg at a time) were separated on 8–12% sodium dodecyl sulphate–polyacrylamide gel electrophoresis (SDS-PAGE) gels and then transferred to PVDF membranes (Millipore, Billerica, MA, USA) in an ice bath. The membranes were incubated in a solution containing 5% skimmed milk TBST following a one-hour incubation time at room temperature. The mixture was then incubated at 4°C for the entire night after the addition of ITIH2 antibody (Cat. #DF13454; Affinity Biosciences LTD, Cincinnati, OH, USA) and β-actin antibody (Cat. # AF7018; Affinity Biosciences LTD, Cincinnati, OH, USA). The next day, the goat anti-rabbit antibody (Cat. # S0001; Cincinnati, OH, USA) was diluted to a dilution of 1:3000 and the PVDF membrane was treated three times with TBST before being left to stand at room temperature for an hour. The Fluor Chem M System (Protein Simple, San Francisco, CA, USA) and the enhanced chemiluminescence (ECL) reagent (Millipore) were used to identify protein bands after normalization to β-actin levels. ImageJ software (National Institute of Health, Bethesda, MD, USA) was used for quantification.

### 2.9 Plate cloning

During the logarithmic growth phase, the stably converted cells were trypsinized, enumerated, and resuspended in the whole media. Each group of 1500 cells was injected into a six-well plate. The cell culture was continued by incubating the six-well plate at 37 °C with 5% CO_2_ after adding 2 ml of complete media to fully mix the cells. The culture was kept for two to three weeks. The six-well plate should be placed under an inverted white light microscope in order to count clone patches that contain more than 50 cells. After that, photograph the clones for future reference.

### 2.10 Cell viability CCK‑8 assay

Changes in cell viability were measured using the Cell Counting Kit-8 (CCK8). Stabilized transformed CRC cells were specifically seeded onto 96-well plates at a density of 3000 cells/well in 100µl of complete medium 24 hours after transfection, and they were cultivated at 37°C. At the end of each experiment, 10µl of CCK8 reagent (Beyotime, Shanghai, China) was added to each well. The cells were then cultured for an additional two hours at 37°C. Optical density measurements (OD450) were then determined using a Multiskan microplate analyzer (Thermo Fisher Scientific, Waltham, MA, USA).

### 2.11 Cell Transwell migration and invasion assay

The Transwell experiment used a Transwell chamber (Corning Company, Corning, NY, USA) with an 8µm pore size in a 24-well plate to assess cell migration and invasion capacity. In the cell migration experiment, 1 × 10^4^ cells were seeded in the upper chamber using 200µl of basic media without FBS, and 600µl of complete medium with 15% FBS was sown in the lower chamber. Any cells that remained on top of the filter were scraped off using a cotton swab following a 48-hour incubation period at 37°C. They were examined under a microscope, counted, and photographed in five randomly selected fields of vision (Olympus, Tokyo, Japan).

For the invasion assays, 50 µL of Matrigel (BD Bioscience, San Jose, CA, USA) was applied to the filter beforehand. Following a 1:8 dilution with ice-cold serum-free medium, the Transwell chamber was hydrated for 30 minutes and incubated for three hours at 37°C. The experiment’s remaining steps are identical to those in the tumor cell Transwell migration experiment.

### 2.12 Transcriptome sequencing

After quantitative RT-PCR analysis and western blot confirmed that ITIH2 overexpression and knockdown were successful in SW620 and Caco2 cell lines, these genetically altered cell models were then exposed to transcriptome profiling using RNA sequencing using the Illumina NovaSeq 6000 platform. The cells’ total cellular RNA was separated using the TRIzol reagent, and the resulting RNA quality and concentration were deemed adequate.

#### Library preparation for transcriptome sequencing.

We used magnetic beads with poly-T oligo attachments to purify mRNA. Using divalent cations in first strand synthesis reaction buffer (5x), lysis was performed at elevated temperatures. First-strand cDNA was produced using M-MuLV reverse transcriptase and random hexamer primers, and RNaseH then degraded it. The second strand of cDNA was made using dNTP and DNA polymerase I. The remaining overhangs became blunt ends as a result of the exonuclease/polymerase activity. After the 3′ end of the DNA fragment was adenylated, an adaptor with a hairpin loop structure was ligated and prepared for hybridization. Library fragments were purified using the AMPure XP technology (Beckman Coulter, Beverly, USA) to choose cDNA fragments with lengths ranging from 370 to 420 bp. Following PCR amplification, the PCR products were purified using AMPure XP beads to yield the library. A Qubit 2.0 fluorometer was used to measure the library for the first time after construction was finished. After diluting it to 1.5 ng/μl, the size of the library insert was measured using an Agilent 2100 Bioanalyzer. Once the insert size was as anticipated, the library’s effective concentration (>2nM) was precisely measured using qRT-PCR to guarantee its quality.

#### Transcriptome sequencing.

Following the qualification of the clustering and sequencing libraries, Illumina NovaSeq 6000 was used for sequencing.

#### Data analysis.

DEGs analysis was performed on cells that were successfully overexpressing ITIH2 and control cells using the DESeq2 R program (3.20.0). By employing a model based on the negative binomial distribution, DESeq2 provides statistical methods for identifying differential expression in numerical gene expression data. Significant differential expression is defined as |log2FC| > 1 and *P* < 0.05.

The Kyoto Encyclopedia of Genes and Genomes (KEGG) and Gene Ontology (GO) enrichment analyses of differentially expressed genes were implemented using the clusterProfiler R package (3.14.3). Corrected p-values for differential genes were considered significantly enriched if they were less than 0.05.

The raw transcriptome sequencing data of the overexpression stable cell line were uploaded as supplementary table 3 in S1 File, and those of the knockdown stable cell line were uploaded as supplementary table 4 in S1 File.

### 2.13 Tumor mouse model establishment

This study was carried out in strict accordance with the recommendations in the Guide for the Care and Use of Laboratory Animals of the National Institutes of Health. The protocol was approved by Kunming Medical University’s Animal Ethics Committee (kmmu20241629). All surgery was performed under sodium pentobarbital anesthesia, and all efforts were made to minimize suffering. The total number of BALB/c mice was twenty. The supplier of the experimental mice was Yunnan Bethesda Biological Company (Kunming, China). The BALB/c mice used in the experiment were housed in rooms that were between 26 and 28 degrees Celsius. Relative humidity was kept between 40 and 60 percent. Ten hours of light and fourteen hours of darkness were maintained throughout the day. Water and food were available at all times. The overexpression cell lines were modeled using SW620 cells, while the knockdown cell lines were modeled using HT-29 cells to produce a stable transient strain model. All injection sites were located in the left lower abdominal region of the mice, corresponding to the splenic area. To assess the effect of ITIH2 gene expression on tumor growth, the tumor volume of loaded mice was observed and evaluated. Four-week-old nude mice weighing 15 ± 1.2 g were presented to the SPF experimental environment for a week of adaptive feeding after being randomly assigned to four groups of five (the mice were numbered before being put in random number columns). A cell suspension with a density of 1 × 10^6^ cells/ml was obtained by first extracting and dissolving the cells of the logarithmic growth phase stable transfer strain in PBS buffer. For a while, the cell suspension was kept on ice. Inside the biological safety cabinet, the mice’s skin was sterilized three times using 75% medical alcohol before being injected. Before inoculation, the cell solution was again broken up and mixed, and 0.1 ml of it was injected subcutaneously into the mice using a 1 ml syringe. One milliliter of a 3% pentobarbital sodium solution was injected intraperitoneally for every kilogram of body weight. The solution was prepared in sterile saline. To calculate mouse mortality, we employ the following criteria: the animal is motionless, near death, or does not respond to modest stimulation. breathing problems, typically accompanied by salivation and/or cyanosis of the mouth and nostrils. When mice inoculated with HT-29 and SW620 cells were killed after four weeks, the final volume of mouse tumors was determined. To minimize animal suffering to the greatest extent, we established strict humane endpoint criteria. When any of the following conditions occurred, euthanasia was immediately performed. Tumor burden must not exceed 5% of the animal’s initial body weight; Tumor location or growth must not severely impair normal physiological functions or cause evident distress; Body weight loss exceeding 20% of the initial weight (considering the mass contribution of the tumor itself); Ulceration or infection present at the tumor site; Persistent self-mutilation behavior. All surviving animals were euthanized via carbon dioxide overdose, after which the final volume of liver metastasis tumors was measured.

### 2.14 Immunohistochemistry

Under ethical approval, archival formalin-fixed paraffin-embedded (FFPE) tissue specimens were acquired retroactively from the First Affiliated Hospital of Kunming Medical University’s Department of Pathology. The 60 clinically annotated cases in this study cohort were surgically removed between March 2023 and January 2025, and included 30 patients with original CRC adenocarcinoma and 30 matched cases with synchronous liver metastases (≥3 metastatic foci per case). Diagnosis was confirmed by two independent pathologists following the 9th edition AJCC criteria, with exclusion criteria applied for neoadjuvant therapy recipients. Before being sectioned (4 μm thick) for immunohistochemical examination using an anti-ITIH2 antibody (Affinity, DF13454; 1:200 dilution), tissue blocks were kept at 4°C in nitrogen-sealed containers.

Quantitative immunohistochemical analysis: ITIH2 was shown to be expressed in the cell membrane and cytoplasm throughout this study. The ratio of each cell’s unique hues to its number of colorations was used for semi-quantitative analysis. It was evaluated independently by two Department of Pathology investigators, who then used the Fromowitz criteria to provide a score [8]. Five randomly chosen fields were used to analyze the sections under a 200x orthogonal white light microscope. Together with statistical scores for staining intensity and positive cell staining rate, positive criteria were counted. The percentage of cells with a positive staining rate was determined using the ImageJ software version 1.54 (National Institutes of Health, Bethesda, M D, USA). The mean percentage of positive cells was determined by calculating the percentage of positive cells (the ratio of the number of brown-stained cells to the total number of cells) in three representative images. A represents the proportion of positive cell counts: A ≤ 1%, 0；1% A ≤ 10%, 1；10% A ≤ 50%, 2；51% A ≤ 75%, 3；75% A ≤ 100%, 4. B represents the percentage of staining intensity: Tan, 2; pale yellow, 1; yellow-brown-tan, 3; and no staining, 0 A x B ≥ 2, which denotes a positive CCL5. The score is as follows: negative, 0–2, mildly positive (+), 2–3, moderately positive (++), 3–4, very positive (++++, ++++), 5–12 [9].

### 2.15 ELISA

Incubation and Sample Addition: Standard wells: Gradient-diluted ITIH2 standards (0, 1, 5, 10, 50, 100 ng/mL) should be added in 100 μL/well. Sample wells: Fill each well with 100 μL of diluted serum. PBS buffer is used for negative control wells. After two hours of incubation at 37°C, wash three times. Incubate 100 μL/well with biotin-labeled anti-ITIH2 antibody (1:1000 dilution) for one hour at 37°C, followed by three washes for detection antibody incubation. The Enzyme Conjugate Reaction involves adding 100 μL of streptavidin-HRP (1:5000 dilution) per well, incubating for 30 minutes at 37°C in the dark, and then washing five times. Color Development and Termination: Add 100 μL of TMB substrate per well, and after 15–20 minutes of dark incubation, the color changes blue. When 50 μL of stop solution (2M H₂SO₄) is added per well, the color turns yellow. Detection: Use a microplate reader to measure OD values at 450 nm, using 630 nm as the reference wavelength for adjustment. Plot a standard curve (four-parameter logistic fit) using the x-axis for standard concentrations and the y-axis for OD values to analyze the data. To determine the actual content (ng/mL), multiply the ITIH2 concentration by the dilution factor after calculating it using the sample OD values.

### 2.16 Statistical data analysis

The two independent samples t-test was used to statistically examine comparisons between the mouse groups. The experimental procedure is homogeneous, we employ randomized groups, and the data results are typically regularly distributed. A two-sided test was used to examine all of the research, and a difference was deemed statistically significant when *P* < 0.05.

## 3 Results

### 3.1 Dataset analysis identifies conserved metastasis-associated DEGs

**Fig 1:**
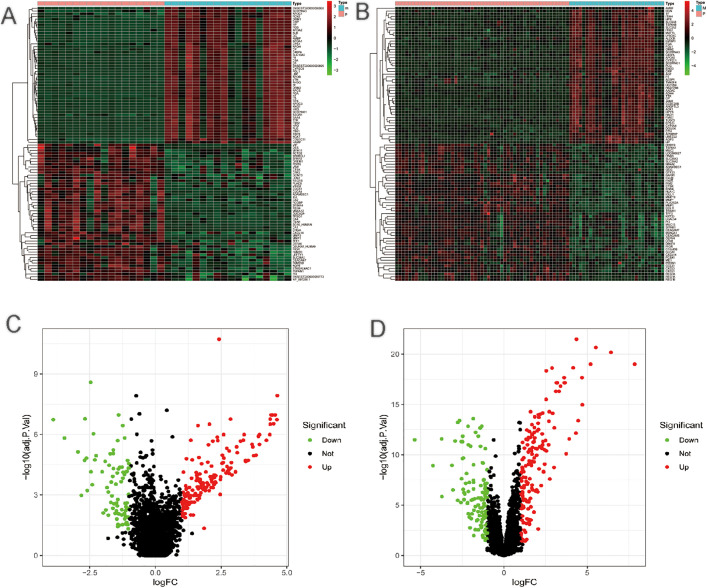
Differential Gene Expression Analysis. **(A)** and **(B)** Heatmaps of the top 100 DEGs from the GSE6988 and GSE14297 datasets. Respectively. Black squares stand for genes that have not changed, pink for primary CRC, and blue for metastatic CRC. Green squares show genes that are down-regulated, and red squares show genes that are up-regulated. **(C)** and **(D)** display volcano plots of all DEGs identified in the GSE6988 and GSE14297 datasets, respectively; unmodified genes are indicated by black circles, down-regulated genes are indicated by green circles, and up-regulated genes are represented by red circles.

Using their special paired primary-metastasis sample design to reduce patient-specific noise, we conducted a cross-dataset analysis of GSE14297 and GSE6988 to find genetic drivers conserved across CRC liver metastases. In GSE6988 and GSE14297, this method produced 291 and 240 DEGs, respectively (Fig 1). The significant quantity of DEGs highlights the widespread transcriptional reprogramming linked to metastasis. The top 100 most significantly changed genes from each dataset are shown in Figs 1A and 1B for clarity, displaying both common and distinct expression patterns.

### 3.2 WGCNA pinpoints clinically relevant co-expression modules

**Fig 2:**
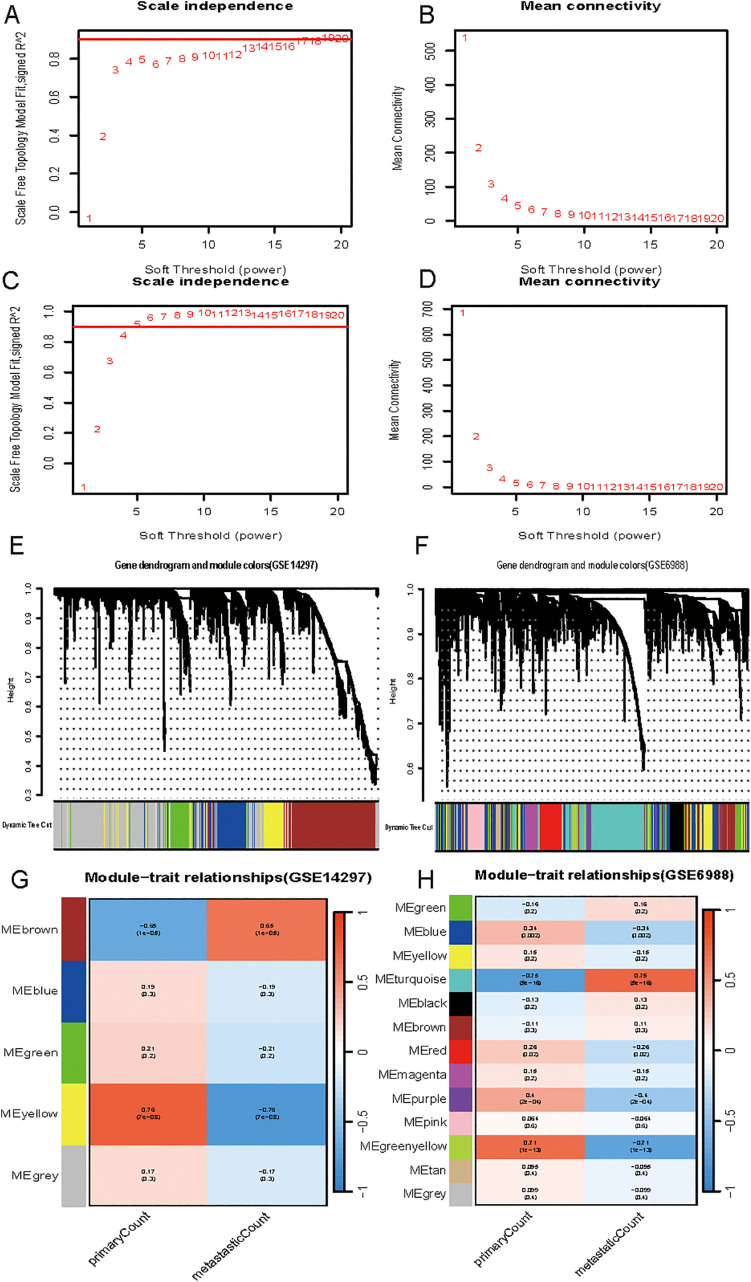
Identification of Differentially Expressed Genes Using WGCNA. **(A)**, **(B)**, **(C)** and **(D)** display network topology analysis for various soft-threshold powers for the GSE6988 and GSE14297 datasets, respectively. examining the scale-free topology. The adjacency matrix is defined by soft thresholding. The GSE6988 and GSE14297 datasets’ respective WGCNA clustering dendrogram and module assignments are displayed in **(E)** and **(F)**. A branch is a cluster of genes that are closely related to one another. The colors of the horizontal bars match those of the modules. Gene clustering modules for differential genes between colorectal cancer liver metastatic tumors and beginning colorectal cancer are shown in **(G)** and **(H)** of the GSE6988 and GSE14297 datasets, respectively.

Using the WGCNA approach, we generated co-expression networks and modules of DEGs between primary CRC and CRC liver metastatic tumors between the GSE14297 and GSE6988 datasets. Figs 2A, 2B,2C and 2D illustrate the R^2^ = 0.97 and R^2^ = 0.97 of the scale-free topology utilized for the GSE6988 and GSE14297 datasets, respectively. A reinforced neighbor matrix was created from the Pearson correlation matrix of the DEGs between the two datasets. All of the selected genes were clustered using a dissimilarity metric based on the topological overlap matrix (TOM), which is based on a dynamic tree-cutting technique that divides the tree into different color-labeled modules. This is shown in Fig 2E and Fig 2F. Figs 2G and 2H show the co-expression modules for each gene. We select the modules having the strongest positive connection for additional analysis. In the GSE14297 dataset, the strongest positive association is found between the brown and yellow modules. The greenyellow and turquoise modules in the GSE6988 dataset had the strongest positive associations.

### 3.3 Identification of DEGs and PPI network construction of DEGs

**Fig 3:**
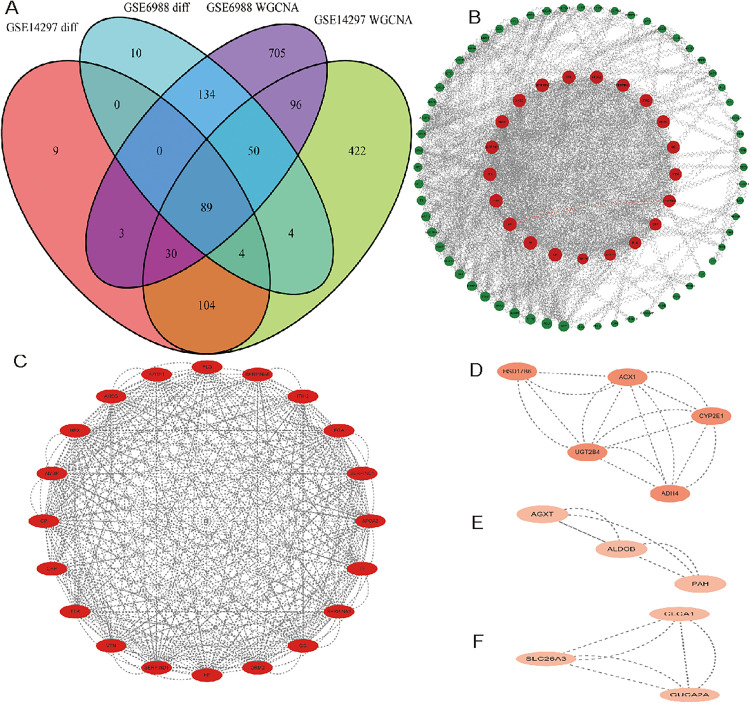
Hub genes linked to metastases are identified using multi-omics integration. **(A)** Venn analysis of differential expression showed that the GSE14297 and GSE6988 datasets, respectively, contained 240 and 291 DEGs (|log2FC| > 1, FDR < 0.05). WGCNA identified 1,107 (blue module) and 801 (turquoise module) genes linked to metastases. 89 consensus genes were obtained using intersection **(B)** CytoHubba-ranked PPI network topology (node degree≥15). MCODE-identified molecular complexes **(C–F)**, where betweenness centrality is reflected in the node coloring.

We used the intersection of the most relevant genes of the modules found in Result 2 and the DEGs found in Result 1 to calculate the final 89 DEGs. The results are presented in Fig 3A.

We loaded the 89 DEGs mentioned above into the STRING database in order to identify the gene interactions. Using the Cytoscape plug-in cytoNCA, the PPI network was analyzed to identify hub genes. (Fig 3A). CP(22 nodes), TF(19 nodes), ORM2(20 nodes), HP(27 nodes), FGA(33 nodes), HPX(22 nodes), SERPINC1(34 nodes), AMBP(33 nodes), AHSG(36 nodes), SERPINA3(22 nodes), TTR(31 nodes), APOA2(31 nodes), SERPIND1(24 nodes), ITIH2(20 nodes), GC(26 nodes), VTN(26 nodes), SERPINA6(15 nodes), CRP(25 nodes), PLG(30 nodes), and AZGP1(16 nodes) were among the top 20 genes found to be potential hub genes based on the LAC algorithm. Meanwhile, to identify important gene cluster modules and offer cluster scores, we analyzed the PPI network using Cytoscape’s MCODE plugin (filter criteria: degree cut-off = 2; node score cut-off = 0.2; k-core = 2; max depth = 100). Four modules were acquired (Figs 3B-3F). PLG, AZGP1, AHSG, HPX, AMBP, CP, CRP, TTR, VTN, SERPIND1, HP, ORM2, GC, SERPINA3, TF, APOA2, SERPINC1, FGA, ITIH2, and SERPINA6 were among the top 20 genes that we examined for potential hub genes. When we combined the top 20 genes from the two algorithms, we found that the hub genes obtained by MCODE and cytoNCA fully overlapped. The results are presented in Figs 3B-3F.

### 3.4 Transcriptome profiling of ITIH2-modified cell lines

**Fig 4:**
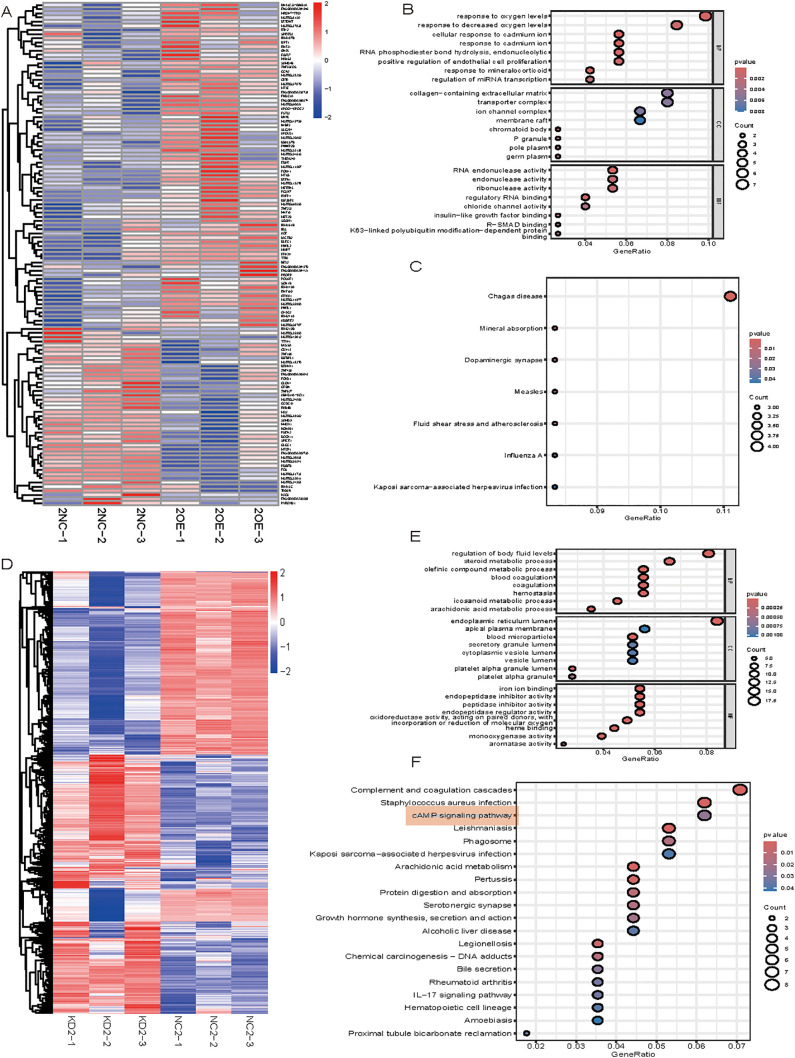
Transcriptome analysis of cellular processes regulated by ITIH2. **(A, D)** Gene heatmaps showing differential expression in ITIH2-knockdown Caco2 and ITIH2-overexpressing SW620, respectively. Expression levels are indicated by a color scale: blue indicates downregulation, and red indicates upregulation. **(B, E)** GO enrichment study of cellular components (CC), molecular functions (MF), and biological processes (BP) for ITIH2-knockdown (E) and ITIH2-overexpressing (B) models. **(C, F)** ITIH2-overexpressing (C) and ITIH2-knockdown (F) models’ KEGG pathway enrichment analysis. The size of the circle represents the number of genes per phrase, while the intensity of the color indicates the statistical significance of the enrichment.

We used transcriptome sequencing on modified cell lines to interpret the functional effects of ITIH2 modification. A high enrichment for processes associated with cellular stress response (e.g., response to oxygen, cadmium ions) and extracellular matrix architecture was found by GO/KEGG analysis of genes dysregulated by ITIH2 overexpression in SW620 cells (Fig 4). On the other hand, ITIH2 knockdown in Caco2 cells changed genes related to lipid and steroid metabolism. While its absence upsets metabolic homeostasis, these conflicting indications imply that ITIH2 may promote adaptability to microenvironmental stressors and modify the extracellular landscape, hence facilitating metastasis.

### 3.5 ITIH2 drives aggressive phenotypes *in vitro* and *in vivo*

The pro-metastatic function of ITIH2 was then functionally confirmed by a variety of gain- and loss-of-function tests.

**Fig 5:**
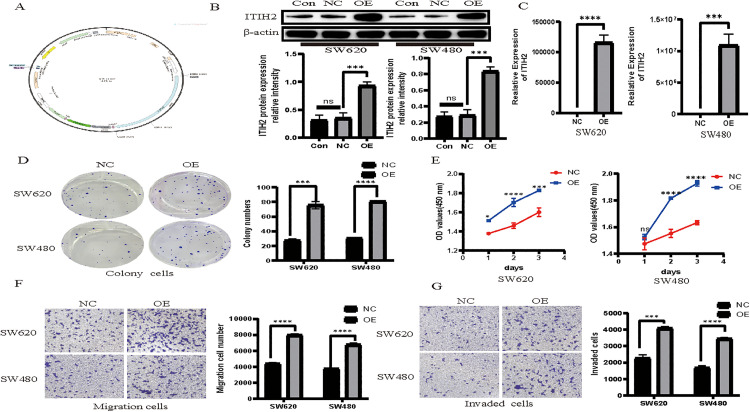
ITIH2 overexpression facilitates the proliferation, migration, and invasion of CRC cells *in vitro.* In the CCK-8 experiment, the cloning ability of SW620 and SW480 increased 24 and 48 hours after ITIH2 overexpression, respectively; **(A)**: The human ITIH2 gene was overexpressed in a viral vector; **(B)** and **(C)** qRT-PCR and western blot showed that SW620 and SW480 were successfully overexpressed by the created ITIH2; **(D)** Plate cloning formation tests and quantitative data showed that the cloning ability of SW620 and SW480 was enhanced following ITIH2 overexpression; **(E)**Transwell tumor cell migration experiment demonstrate that the migration capabilities of SW620 and SW480 increased after ITIH2 overexpression; quantitative data and experiment **(F)** demonstrate the invasion capabilities of SW620 and SW480. ns, no significance; ** *P* < 0.01; *** *P* < 0.001; **** *P* < 0.001.

**Fig 6:**
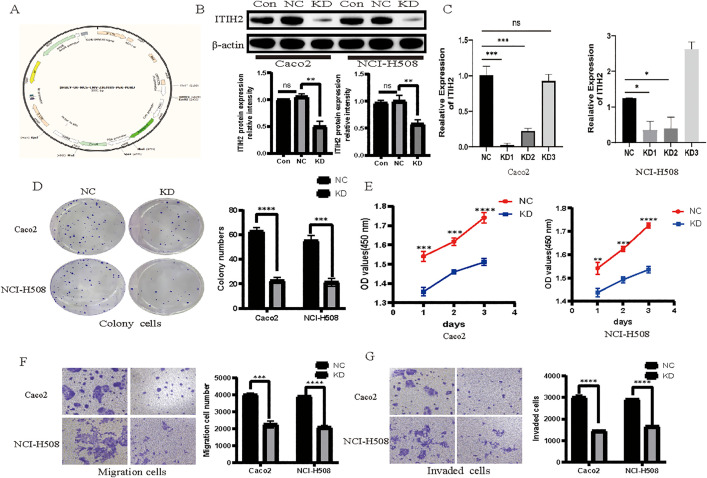
*In vitro* migration, invasion, and proliferation of CRC cells are all inhibited by ITIH2 knockdown. **(A)** viral vector was created that knocked down the human ITIH2 gene; **(B)** and **(C)** qRT-PCR and western blot verified that the ITIH2 knocked down Caco2 and NCI-H508 successfully; **(D)** plate cloning formation experiments and quantitative data demonstrated that the cloning ability of Caco2 and NCI-H508 declined following ITIH2 knockdown. **(E)** In the CCK-8 experiment, Caco2 and NCI-H508’s capacity to proliferate declined 24 hours following ITIH2 knockdown; **(F)** Quantitative data from a Transwell tumor cell migration experiment demonstrated that Caco2 and NCI-H508’s capacity to migrate decreased following ITIH2 knockdown; **(G)** Quantitative data from a Transwell tumor cell invasion experiment demonstrated that Caco2 and NCI-H508’s capacity to invade decreased following ITIH2 knockdown.

Oncogenic Properties *in Vitro*: SW620 and SW480 cells’ ability to proliferate, migrate, and invade was markedly increased by ectopic expression of ITIH2 (Figs 5D-5G). ITIH2 knockdown, on the other hand, had the opposite impact in Caco2 and NCI-H508 cells, effectively suppressing these cancerous tendencies (Fig 6).

**Fig 7:**
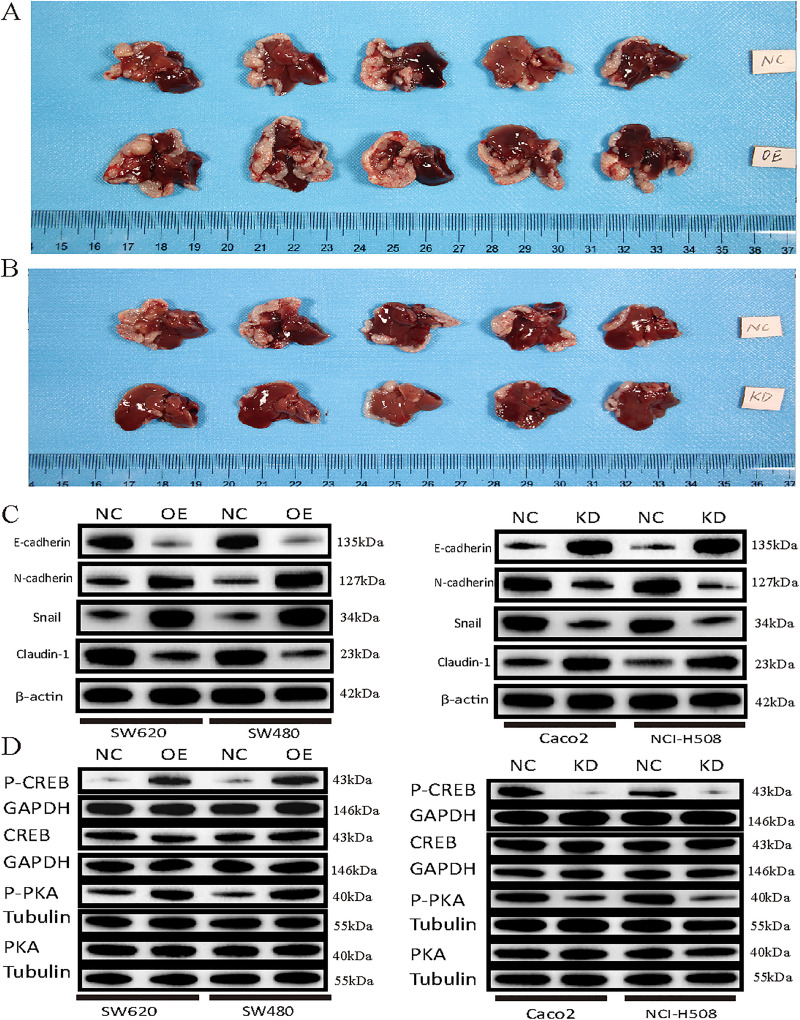
Effects of ITIH2 overexpression and knockdown on tumor growth and protein expression in vivo and in vitro. **(A)** Quantitative data results and tumor volume changes in mice after ITIH2 gene overexpression; **(B)** Quantitative data results and tumor volume changes in mice after ITIH2 gene knockdown; **(C)** EMT-related protein alterations following ITIH2 overexpression and knockdown were detected using Western blot; (D) as were changes in proteins linked to the cAMP-PKA-CREB signaling pathway following ITIH2 overexpression and knockdown.

*In Vivo* Tumor Growth: In mice xenograft models, ITIH2’s pro-tumorigenic action was validated. While ITIH2 knockdown resulted in a similar 1.9-fold decrease in tumor size (Fig 7B), ITIH2 overexpression accelerated tumor growth and produced a 1.9-fold greater endpoint tumor volume (Fig 7A). Our in vitro results are strongly supported by these in vivo data, which demonstrate that ITIH2 is a strong promoter of CRC tumor growth.

### 3.6 ITIH2 promotes metastasis via EMT and interaction with key oncogenic pathways

**Fig 8:**
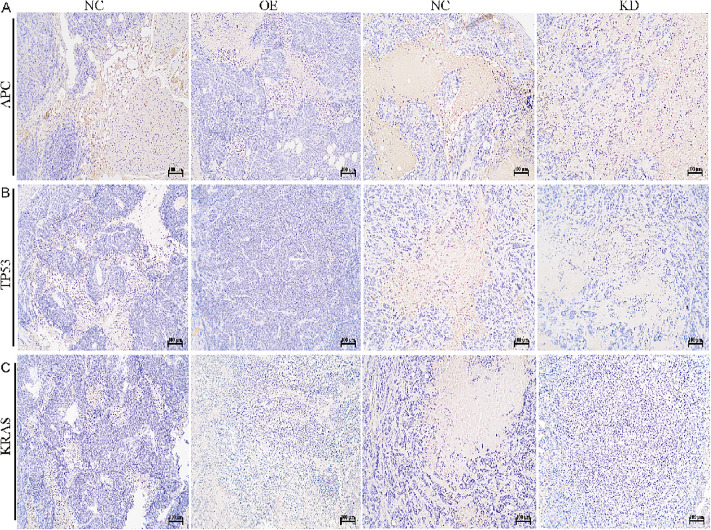
The prognostic value of ITIH2 has been validated clinically. **(A)** representative immunohistochemical staining of ITIH2 in primary CRC versus liver metastasis tissues; **(B)** semi-quantitative IHC-score comparison between clinical subgroups; **(C)** Elisa found that patients with colorectal cancer and those with liver metastases had ITIH2 expression in their blood. **(D)**; **(E)** The TCGA cohort’s Kaplan-Meier overall survival curves stratified by ITIH2 expression levels; **(F)** disease-free survival analysis based on ITIH2 expression profiling via GEPIA2 platform.

We looked at ITIH2’s connection to the epithelial-mesenchymal transition (EMT), a key component of metastasis, in order to better understand the processes by which it promotes metastasis. ITIH2 overexpression triggered a complete EMT program, but its knockdown corrected this phenotype, according to Western blot analysis (Fig 7C). Additionally, immunohistochemistry on xenograft tissues revealed a possible interaction between ITIH2 and important CRC drivers: overexpression of ITIH2 was linked to simultaneous elevation of KRAS and downregulation of TP53 and APC (Fig 8). This trend suggests that some oncogenic alterations, especially KRAS activation, may enhance ITIH2’s pro-metastatic effect.

### 3.8 Clinicopathological correlation of ITIH2 expression

**Fig 9:**
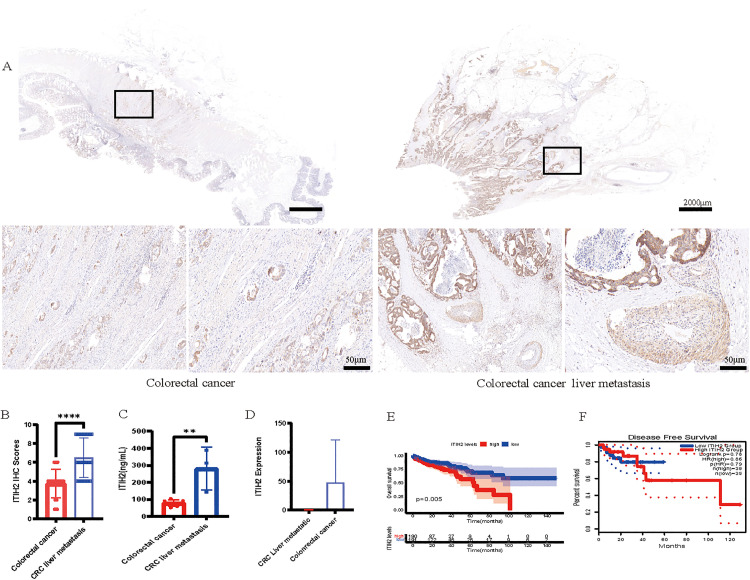
Correlation of gene expression levels among KRAS, TP53, APC, and ITIH2. **(A)** Changes in APC expression after knockdown and overexpression of ITIH2; **(B)** Changes in TP53 expression after knockdown and overexpression of ITIH2; **(C)** Changes in KRAS expression after knockdown and overexpression of ITIH2.

Using validated anti-ITIH2 antibody, tissue microarrays including 30 primary CRC and 30 corresponding liver metastases specimens were subjected to immunohistochemical (IHC) profiling. When compared to primary tumors, metastatic lesions showed noticeably higher ITIH2 expression, according to semi-quantitative IHC-score analysis (Figs 9A and 9B). ITIH2 expression levels in the blood of patients with primary CRC and those with liver metastases from CRC were found using ELISA. Fig 9C illustrates the findings, which showed a substantial rise in ITIH2 levels in the blood of individuals with liver metastases from CRC. In the external dataset GSE92914, the expression of ITIH2 in liver metastases was significantly higher than that in primary CRC (Fig 9D).

By utilizing Kaplan-Meier survival curves with log-rank testing to analyze CRC transcriptome profiles obtained from The TCGA database (Project ID: TCGA-COAD), we assessed the predictive significance of ITIH2 expression. A univariate Cox proportional hazards analysis showed a significant correlation between worse OS and increased ITIH2 expression (dichotomized by median cutoff). ITIH2 expression levels and disease-free survival, however, did not significantly correlate (Figs 9E and 9F). We also assessed the clinical relevance of ITIH2 expression in patients with rectal cancer. Research revealed that in patients with rectal cancer, there was no correlation between ITIH2 expression and OS or DFS. The experimental results are shown in Supplementary Figure 2 in S1 File. The prognostic analysis results for the remaining genes are presented in Supplementary Figures 3 and 4 in S1 File, all of which yielded negative findings.

## 4 Discussion

According to our research, ITIH2 functions as a hub gene in the occurrence and progression of CRC liver metastasis and may be involved in the process of liver metastasis in CRC. This suggests that ITIH2 may be a viable therapeutic target for the treatment of liver metastases from CRC. Furthermore, our results indicate that a poor prognosis for CRC is linked to high expression of ITIH2. An essential metric for determining the survival rate of patients with CRC may be the expression level of ITIH2. ITIH2 overexpression dramatically increases CRC cells’ capacity for invasion, migration, and proliferation, whereas ITIH2 knockdown has the opposite effect. In conclusion, this study offers potential treatment targets and assessment techniques for liver metastases of CRC. The experimental methods used in this investigation were chosen to confirm directly that ITIH2 promotes metastasis. For example, to specifically analyze ITIH2’s function in cell migration and invasion, the Transwell and Matrigel invasion assays were selected. We used bulk RNA-seq to fully profile the transcriptome changes following ITIH2 silencing because it provides an objective, worldwide perspective of gene expression, overcoming the shortcomings of microarrays in terms of identifying new transcripts and offering a greater dynamic range.

ITIH proteins have been implicated in a number of pathophysiological processes, such as inflammation and carcinogenesis, according to recent research [10,11]. Depending on the clinical environment, these proteins are either up- or down-regulated; nonetheless, there is strong evidence that all members of the ITIH family are important for tumor growth and cellular malignancy [12,13]. Among these, inter-alpha-trypsin inhibitor heavy chain 2 (ITIH2) has been found to be a putative core gene in individuals with CRC liver metastases, indicating that it may play a role in the process [14]. A structurally related family of plasma serine protease inhibitors implicated in extracellular matrix interactions and structural maintenance includes ITIH2 [15]. The ITIH protein family is essential to the function of hyaluronic acid, which is found in the extracellular matrix and regulates a number of basic biological processes, including tissue morphogenesis, cell migration, and cell proliferation [15]. Several malignancies, such as glioblastoma multiforme and pancreatic cancer, have differential expression of ITIH2. ITIH2 has been found to be a possible diagnostic marker for pancreatic cancer [16].ITIH2 may be linked to tumor differentiation because it is normally produced in low-grade central nervous system cancers and normal brain tissue, but it is not present in high-grade tumors of the central nervous system, such as glioblastoma multiforme [17]. Furthermore, because ITIH2 has an estrogen-binding domain, which may be essential for metastasis and tumor growth given estrogen’s significant influence on extracellular matrix integrity, there is a strong correlation between ITIH2 expression levels and estrogen levels [18]. This may be one way that ITIH2 aids in the development of tumors. The metastatic process should be clarified and eventually addressed with the aid of additional study on ITIH2’s function in tumor growth and metastasis. ITIH2’s role in CRC liver metastasis is still unknown, and there is currently little research on the subject. Therefore, further research is required to identify its methods of action.

Serine protease proteins with a light chain and one or two heavy chains make up the inter-alpha-trypsin inhibitor (ITIH) family [19]. While the heavy chains (encoded by ITIH1, ITIH2, ITIH3, ITIH4, and ITIH5) exist in five different homologs, each encoded by a different gene, the light chain is represented by a single kind, expressed by the AMBP protein. These protein families take part in extracellular structural remodeling, which is essential for cell migration and malignant processes [20]. The expression levels of each ITIH family member vary depending on the type of tumor [13]. Different tumors or disorders may exhibit varying amounts of expression for the same protein. Inflammatory responses are influenced by ITIH2, often referred to as SHAP, which is found close to the 302 kb telomere and forms complexes with hyaluronic acid (HA) [21]. Higher levels of ITIH2 expression are linked to worse patient outcomes in certain disorders, which is consistent with our findings. ITIH2 has been found to be a key gene in the liver metastases of colorectal cancer, and overexpression of this gene is associated with a decreased chance of survival [14]. Another key gene in metastatic uveal melanoma is ITIH2 [22]. A poor prognosis for endometrial and ovarian malignancies is indicated by higher expression levels of ITIH2, which forms complexes with HA [21]. Moreover, ITIH2 is important for the development of multiple myeloma [23]. ITIH2 has a high mutation rate in lung cancer, according to a prior related study, which may indicate that it plays a role in the formation of lung cancer [24]. Obese dogs have higher serum ITIH2 levels linked to metabolic dysfunction than dogs without metabolic dysfunction [25]. ITIH2 levels are considerably higher in patients with more advanced end-stage renal illness than in others, and it may even be a possible indicator of inflammatory reactions [26]. Similar to our findings, which also show that ITIH2 can aid in the progression of CRC, these data collectively imply that ITIH2 is engaged in disease progression and promotes disease development.

Low ITIH2 expression has been linked to a worse prognosis in various cancers, which runs counter to the data above. ITIH2 levels were shown to be considerably greater in low-grade tumors and normal brain tissue than in high-grade gliomas in an examination of primary human brain tumors, suggesting a negative connection with malignancy [27]. Furthermore, glioma cells with persistent overexpression of ITIH2 dramatically suppressed cell proliferation, promoted cell-cell adhesion, and strongly reduced cancer cell invasion [28]. These findings imply that tumor progression may be connected to reduced ITIH2 expression in brain tumors. ITIH2 expression was reported to be considerably downregulated in a cholangiocarcinoma investigation, and tracking changes in ITIH2 levels may be a predictive indicator for cholangiocarcinoma [29]. These findings show that ITIH2 acts through distinct pathways in different disorders, and that more research is necessary to confirm whether it acts as a tumor suppressor or promoter because its effects may change in different conditions.

Our sequencing data demonstrated that dysregulation of the cAMP signaling system occurs from alterations in ITIH2 expression, indicating that the cAMP pathway may play a role in the formation and incidence of liver metastases from CRC. According to earlier research, cAMP signaling can have both tumor-promoting and tumor-suppressive effects. The proliferation, migration, invasion, and metabolism of cancer cells can all be controlled by the cAMP signaling system [30]. Protein kinase A (PKA), exchange proteins that are directly activated by cAMP, and ion-gated channel proteins are all activated by cAMP as a second messenger [31]. cAMP response element-binding protein (CREB), one of PKA’s target proteins, is a crucial transcription factor that controls the expression of oncogenes including cyclin D1 and c-Jun [32]. Depending on the tumor type and environment, the cAMP–PKA–CREB signaling pathway can have both tumor-promoting and tumor-suppressive effects in cancer. Long-term stimulation of cAMP–PKA–CREB signaling enhances CRC growth and metastasis, according to *in vitro* and *in vivo* research on the disease [33]. The stemness and capacity for metastasis of CRC cells are similarly influenced by the cAMP–PKA–CREB pathway. In particular, CREB inhibitors strengthen irinotecan’s ability to prevent CRC liver metastases. These findings imply that CREB inhibition may be a viable treatment approach for metastatic CRC and emphasize the crucial role that CREB plays in preserving the stemness and metastatic potential of CRC cells [34]. CRC growth can be considerably inhibited by blocking the cAMP–PKA–CREB signaling pathway [35]. These results show that the cAMP–PKA–CREB pathway plays a significant role in tumor promotion in CRC. Future research will examine how ITIH2 affects this pathway and encourages liver metastases from CRC.

Our study unavoidably has limitations, even though we downloaded transcriptome data from several databases and used *in vitro* cell assays to support our hypothesis. It is still unknown how exactly ITIH2 contributes to the liver metastases of CRC. Nonetheless, our research shows that ITIH2 expression levels can be used to accurately predict a patient’s prognosis for CRC. Furthermore, our results point to ITIH2 as a possible therapeutic target for the treatment of liver metastases from CRC. In order to establish ITIH2’s role in increasing CRC liver metastasis and to clarify how ITIH2 contributes to this process, future research will need to conduct extensive *in vitro* and *in vivo* tests. This will provide prospective therapeutic targets for treating patients with CRC liver metastases.

## 5 Conclusion

Compared to individuals with primary CRC, those with liver metastases from CRC exhibit a considerably greater expression level of ITIH2. A poor prognosis is highly correlated with high ITIH2 expression in CRC. ITIH2 overexpression significantly increases CRC cells’ capacity for invasion, migration, and proliferation, whereas ITIH2 knockdown has the opposite impact. ITIH2 may be a viable therapeutic target for the treatment of CRC liver metastases as well as an independent predictive biomarker for OS in patients with CRC.

## Supporting information

S1 File Details of lentiviral vectors and gene sequences for ITIH2 overexpression and knockdown. (ZIP)
