# Supplementary material for: ITIH2 in colorectal cancer metastasis: Weighted Gene Co-expression Network Analysis-guided functional validation
Source: PLoS One. 2026 Feb 5;21(2):e0329719. doi: 10.1371/journal.pone.0329719 (PMC12875447; doi:10.1371/journal.pone.0329719)
Supplement: S1 File — (ZIP) [file pone.0329719.s001.zip › supplement material/supplementary table 1.docx]

**supplementary table 1. Details of lentiviral vectors and gene sequences for ITIH2 overexpression and knockdown.**

**Overexpression of the ITIH2 target gene sequence**

ATGAAAAGACTCACGTGCTTTTTCATCTGCTTCTTTCTTTCTGAAGTATCAGGCTTCGAAATCCCCATAAATGGACTTTCTGAATTTGTAGACTATGAAGATCTTGTGGAACTGGCCCCAGGCAAATTTCAATTGGTGGCAGAGAACCGGAGATATCAGAGAAGCCTTCCAGGAGAATCGGAAGAAATGATGGAAGAGGTTGATCAAGTAACTCTTTATAGCTATAAAGTCCAGTCTACTATTACTTCTCGGATGGCCACCACCATGATCCAGAGCAAAGTGGTGAACAATTCCCCGCAGCCTCAGAATGTCGTGTTTGATGTTCAGATCCCCAAAGGAGCATTCATTTCCAACTTCTCCATGACTGTGGACGGCAAGACATTTAGGAGCTCTATTAAGGAGAAAACTGTGGGCCGAGCTCTTTATGCACAGGCCAGAGCAAAAGGCAAGACGGCTGGCTTGGTGAGGAGCAGCGCTCTTGATATGGAAAACTTCAGAACGGAAGTAAATGTCCTCCCAGGAGCAAAGGTGCAGTTCGAACTTCACTACCAGGAGGTGAAGTGGAGGAAGCTGGGCTCCTATGAGCACAGGATCTATCTGCAACCTGGACGGCTGGCCAAACACTTAGAGGTAGATGTGTGGGTTATCGAACCACAGGGACTGAGATTTCTTCATGTTCCCGACACATTTGAAGGCCATTTCGATGGTGTTCCGGTCATTTCTAAAGGACAACAGAAGGCGCACGTCTCCTTCAAGCCCACGGTAGCACAGCAGAGAATATGCCCTAACTGCCGGGAGACTGCGGTAGATGGGGAACTGGTGGTGCTGTATGACGTGAAAAGAGAAGAGAAGGCTGGTGAACTGGAGGTGTTTAATGGATATTTTGTCCACTTCTTTGCTCCTGACAACCTGGACCCAATTCCCAAAAACATCCTCTTTGTCATCGATGTGAGTGGCTCCATGTGGGGAGTTAAAATGAAACAAACTGTGGAAGCAATGAAGACCATATTGGATGACCTCAGAGCAGAAGACCATTTCTCTGTGATTGATTTCAACCAGAACATTCGAACTTGGAGAAATGATTTAATTTCAGCTACAAAAACACAGGTTGCAGATGCCAAGAGGTATATTGAGAAAATCCAGCCCAGTGGAGGCACAAACATCAACGAAGCACTCCTACGGGCAATCTTCATTTTGAATGAAGCCAATAACTTGGGACTGTTAGACCCCAACTCCGTCTCGCTGATCATTTTGGTTTCTGATGGAGATCCAACAGTGGGCGAACTAAAACTGTCAAAAATTCAGAAAAACGTTAAGGAGAACATCCAAGACAATATCTCCTTGTTCAGTTTGGGCATGGGATTTGATGTGGACTATGATTTTTTGAAGAGACTGTCCAATGAAAACCATGGAATTGCACAAAGGATTTATGGAAACCAGGACACGTCTTCCCAGCTTAAGAAATTCTACAACCAGGTCTCCACTCCATTGCTCCGGAATGTTCAGTTCAACTATCCCCATACATCAGTCACGGACGTCACTCAAAACAATTTCCATAACTACTTTGGAGGCTCAGAGATTGTGGTGGCAGGAAAATTTGACCCTGCTAAATTGGATCAAATAGAGAGCGTTATCACGGCGACTTCGGCTAACACGCAGTTAGTCTTGGAGACCCTGGCCCAGATGGACGACTTGCAGGATTTTCTATCGAAAGACAAGCATGCAGATCCCGATTTCACCAGGAAACTGTGGGCCTATCTAACCATCAACCAACTGCTAGCTGAACGAAGCCTGGCTCCTACAGCTGCCGCCAAGAGAAGAATTACAAGATCGATCCTGCAGATGTCTCTAGACCACCACATTGTGACTCCGCTGACCTCGCTGGTGATCGAGAACGAGGCTGGGGATGAGCGCATGCTGGCGGATGCCCCACCGCAGGATCCCTCCTGCTGCTCAGGGGCCCTGTATTACGGCAGCAAAGTGGTTCCAGATTCCACCCCGTCTTGGGCCAATCCTTCACCAACGCCCGTGATCTCCATGCTGGCACAAGGATCTCAGGTGCTAGAGTCCACGCCACCCCCACATGTGATGAGAGTTGAAAATGACCCACATTTCATCATTTATCTACCAAAAAGCCAAAAGAACATTTGTTTCAATATTGACTCAGAACCTGGAAAAATCCTCAACCTGGTTTCTGACCCAGAATCAGGAATTGTAGTCAACGGTCAGCTTGTTGGTGCCAAGAAGCCCAACAATGGAAAACTAAGCACCTATTTTGGAAAACTGGGATTTTATTTCCAAAGTGAAGACATAAAAATAGAAATCAGCACTGAGACCATCACCCTGAGCCATGGTTCTAGCACATTCTCCTTGTCCTGGTCCGACACGGCTCAAGTCACGAATCAGAGGGTGCAGATCTCAGTGAAGAAAGAAAAAGTGGTAACTATCACCCTGGATAAAGAGATGTCCTTTTCTGTTTTACTTCATCGTGTTTGGAAGAAGCATCCCGTCAATGTTGACTTTCTGGGAATCTACATACCCCCTACAAACAAGTTCTCACCTAAAGCCCACGGACTAATAGGCCAGTTCATGCAGGAACCAAAGATACACATCTTCAATGAGAGACCAGGAAAGGACCCTGAGAAGCCAGAGGCCAGCATGGAAGTGAAGGGGCAGAAGCTGATCATCACCAGGGGCTTACAGAAAGACTACAGAACGGATCTAGTGTTTGGAACGGACGTTACCTGCTGGTTTGTGCACAACAGTGGAAAAGGATTCATTGACGGGCATTACAAGGATTACTTCGTGCCTCAGCTCTACAGCTTTCTCAAACGGCCTTAA

**The siRNA and shRNA sequences of the control viral vector are as follows:**

siRNA：TTCTCCGAACGTGTCACGTAA

shRNA：

Top strand: GATCCGTTCTCCGAACGTGTCACGTAATTCAAGAGATTACGTGACACGTTCGGAGAATTTTTTC

Bottom strand: AATTGAAAAAATTCTCCGAACGTGTCACGTAATCTCTTGAATTACGTGACACGTTCGGAGAACG

siRNA1：GGCAAGACATTTAGGAGCTCTATTA

siRNA2：CAGGTTGCAGATGCCAAGAGGTATA

siRNA3：CATTGCTCCGGAATGTTCAGTTCAA

|  | Top strand | Bottom strand |
| --- | --- | --- |
| shRNA1 | GATCCGGCAAGACATTTAGGAGCTCTATTACTCGAGTAATAGAGCTCCTAAATGTCTTGCCTTTTTTG | AATTCAAAAAAGGCAAGACATTTAGGAGCTCTATTACTCGAGTAATAGAGCTCCTAAATGTCTTGCCG |
| shRNA2 | GATCCGCAGGTTGCAGATGCCAAGAGGTATACTCGAGTATACCTCTTGGCATCTGCAACCTGTTTTTTG | AATTCAAAAAACAGGTTGCAGATGCCAAGAGGTATACTCGAGTATACCTCTTGGCATCTGCAACCTGCG |
| shRNA3 | GATCCGCATTGCTCCGGAATGTTCAGTTCAACTCGAGTTGAACTGAACATTCCGGAGCAATGTTTTTTG | AATTCAAAAAACATTGCTCCGGAATGTTCAGTTCAACTCGAGTTGAACTGAACATTCCGGAGCAATGCG |
