# Supplementary material for: ITIH2 in colorectal cancer metastasis: Weighted Gene Co-expression Network Analysis-guided functional validation
Source: PLoS One. 2026 Feb 5;21(2):e0329719. doi: 10.1371/journal.pone.0329719 (PMC12875447; doi:10.1371/journal.pone.0329719)
Supplement: S1 File — (ZIP) [file pone.0329719.s001.zip › supplement material/supplementary table 2.docx]

supplementary table 2. Primer sequences used for qRT‑PCR.

ITIH2-F GCCATTTCGATGGTGTTCCG

ITIH2-R CGTCATACAGCACCACCAGT

GAPDH-F GGAGTCCACTGGCGTCTTCA

GAPDH-R GTCATGAGTCCTTCCACGATACC
