# Supplementary figures and images for: ITIH2 in colorectal cancer metastasis: Weighted Gene Co-expression Network Analysis-guided functional validation
Source: PLoS One. 2026 Feb 5;21(2):e0329719. doi: 10.1371/journal.pone.0329719 (PMC12875447; doi:10.1371/journal.pone.0329719)

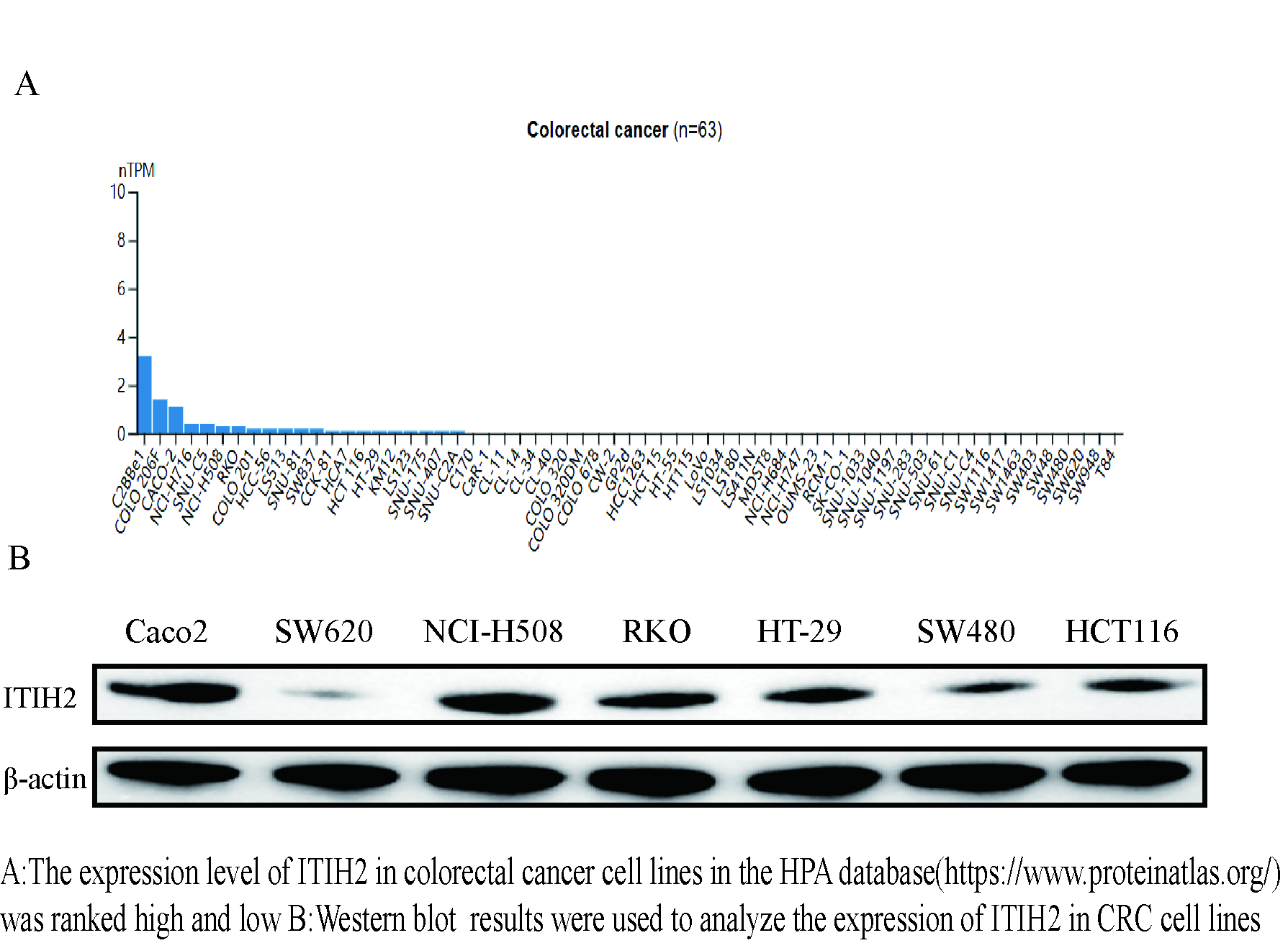

Supplement: S1 File — (ZIP) [file pone.0329719.s001.zip › supplement material/supplementary figure 1.tif]

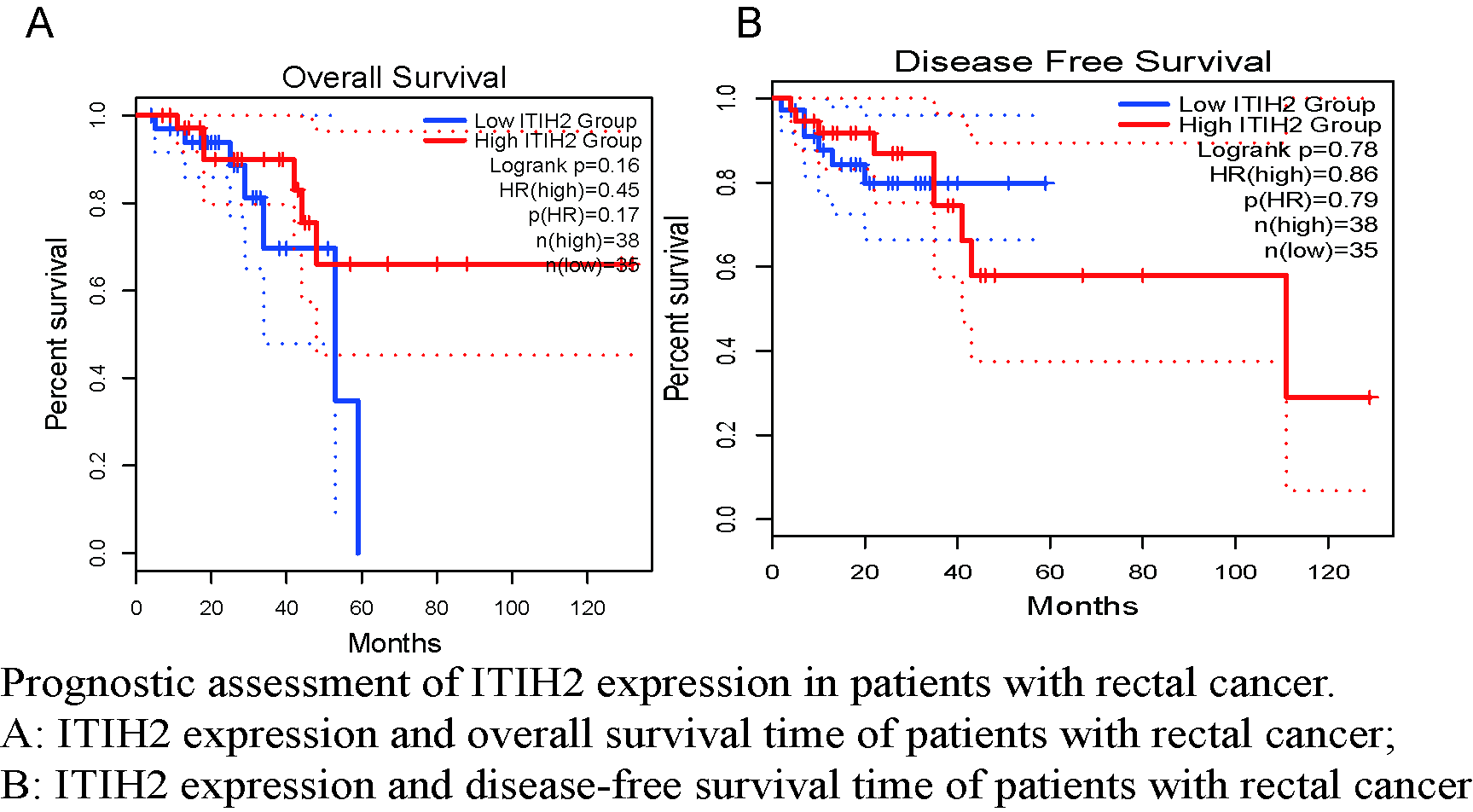

Supplement: S1 File — (ZIP) [file pone.0329719.s001.zip › supplement material/supplementary figure 2.tif]

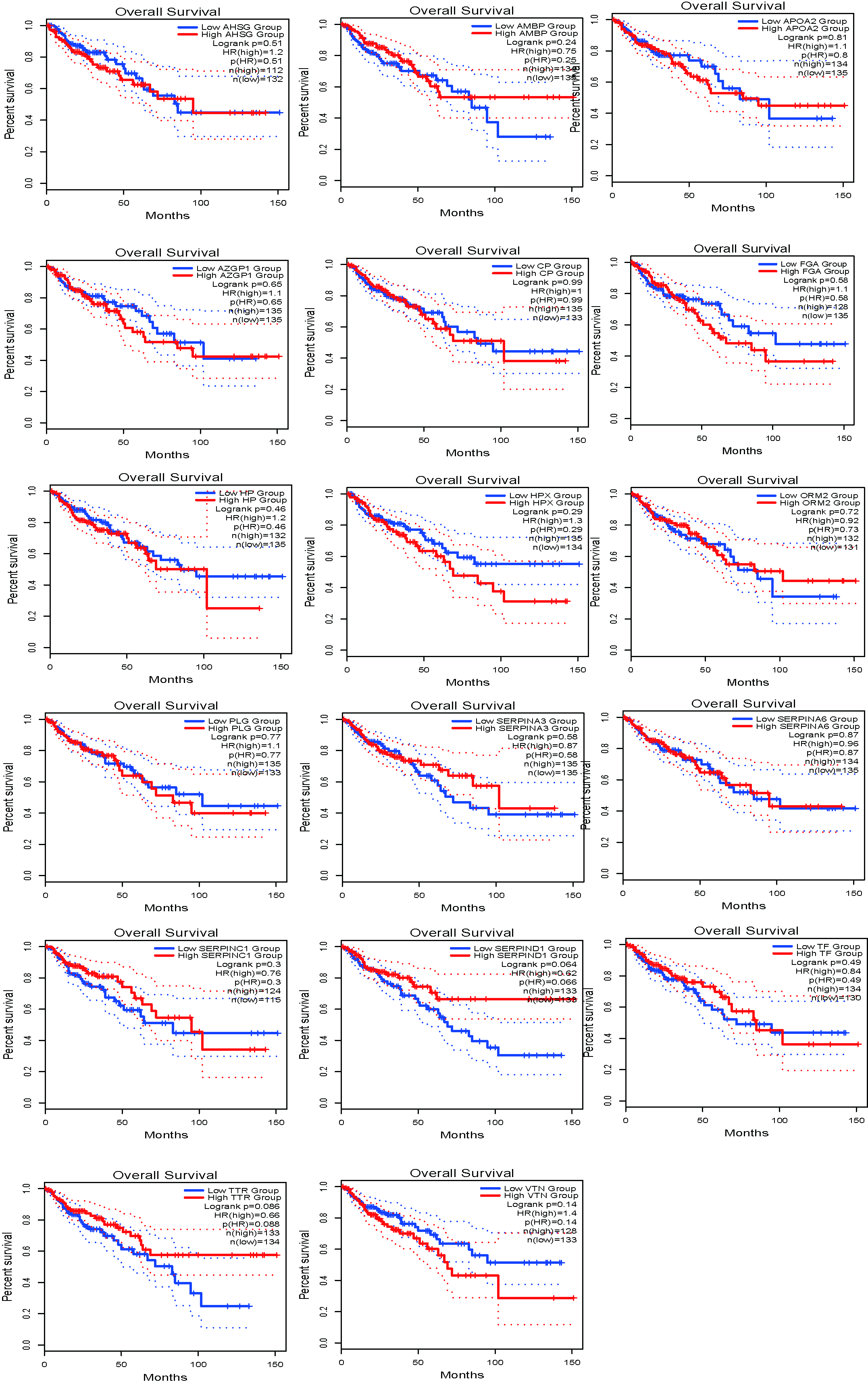

Supplement: S1 File — (ZIP) [file pone.0329719.s001.zip › supplement material/supplementary figure 3.tif]

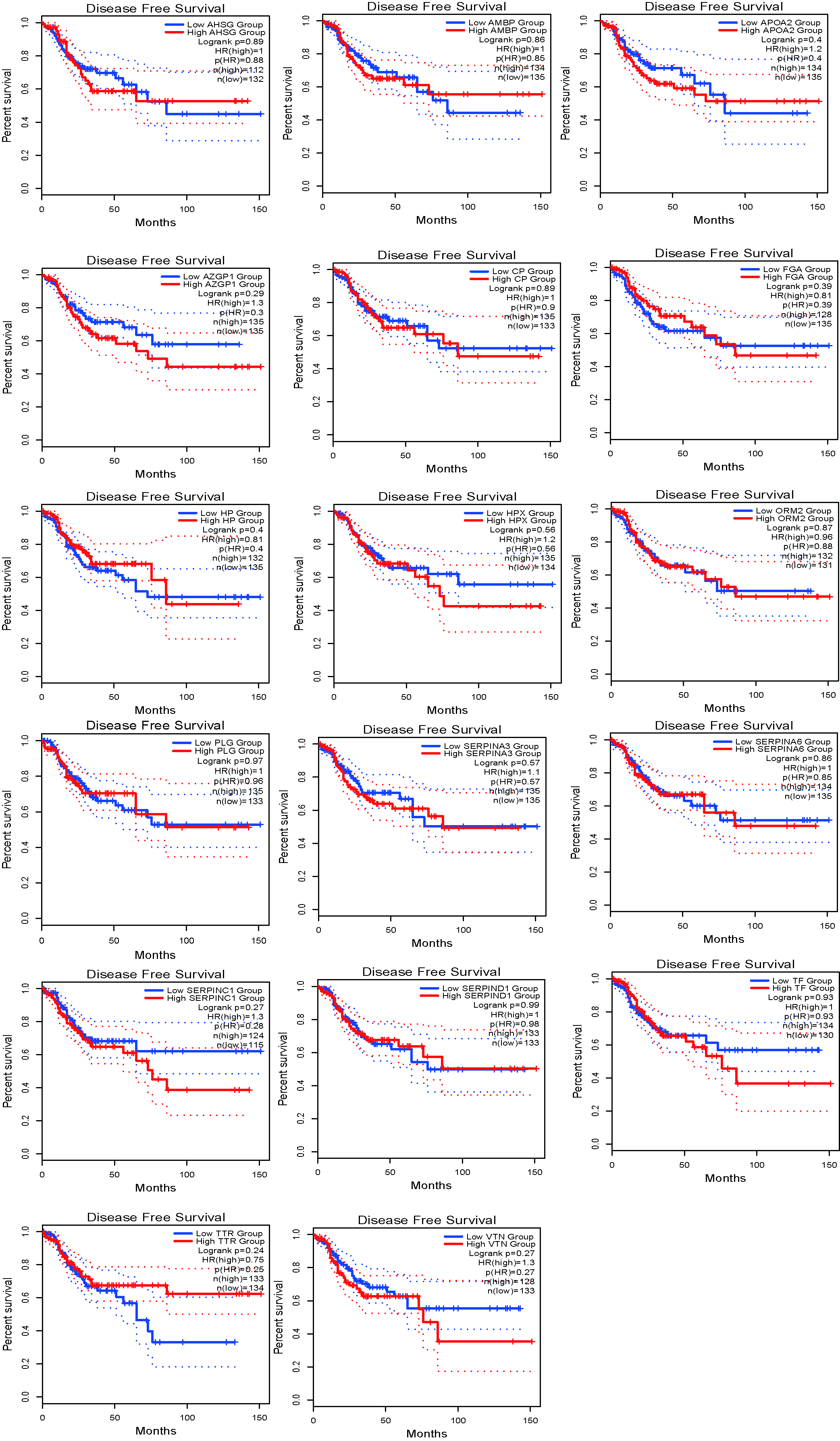

Supplement: S1 File — (ZIP) [file pone.0329719.s001.zip › supplement material/supplementary figure 4.tif]
